# Tincture of Iodine

**Published:** 1844-09-21

**Authors:** 


TINCTURE OF IODINE.

In the Montreal Medical Gazette, (April 1, 1844,) Dr. Crawford draws the attention of the profession to the beneficial influence of Tincture of Iodine (Magendie's form) applied to variola, erysipelas, acute rheumatism, and neuralgia. During the last four years, erysipelas has been very prevalent in Montreal, and Dr. C. has applied the iodine with great apparent advantage. He does not exclude the the use of constitutional remedies; but he has seen the most decided advantages result from the local application, where no internal means were employed. In variola he tried the comparative merits of the iodine and the nitrate of silver, but gives the preference to the former. This application is very manageable and very bearable.—*Med. Chirur. Review.*

